# Diagnostic efficiency and safety of rapid on‐site evaluation combined with CT‐guided transthoracic core needle biopsy in suspected lung cancer patients

**DOI:** 10.1111/cyt.13123

**Published:** 2022-05-10

**Authors:** Ruzetuoheti Yiminniyaze, Xiujuan Zhang, Yuanyuan Zhang, Kun Chen, Chengwei Li, Ning Zhu, Daibing Zhou, Jing Li, Yuhai Zhang, Shengqing Li

**Affiliations:** ^1^ Department of Pulmonary and Critical Care Medicine Huashan Hospital, Fudan University Shanghai China; ^2^ Department of Laboratory Medicine, Baoshan District of Huashan Hospital Fudan University Shanghai China; ^3^ Department of Health Statistics Airforce Medical University Xi'an China

**Keywords:** computed tomography‐guided transthoracic core needle biopsy, diagnostic efficiency, lung cancer, rapid on‐site evaluation, safety

## Abstract

**Objective:**

The efficacy of rapid on‐site evaluation (ROSE) combined with computed tomography‐guided transthoracic core needle biopsy (CT‐guided TCNB) is rarely investigated. This study aimed to evaluate the diagnostic efficiency and safety of ROSE combined with CT‐guided TCNB for suspected lung cancer patients.

**Materials and Methods:**

Clinical data from 285 patients who received CT‐guided TCNB for suspected lung cancer in Huashan Hospital from 2015 to 2018 were retrospectively analysed. Of these 163 patients underwent CT‐guided TCNB combined with ROSE (ROSE group), while the remaining 122 patients underwent without ROSE (non‐ROSE group). The smears from TCNB were quickly processed with Diff‐Quick staining and analysed by a skilled cytologist on‐site. The consistency of ROSE with the final clinicopathological diagnosis and the diagnostic efficiency and safety of ROSE combined with CT‐guided TCNB in suspected lung cancer patients were evaluated.

**Results:**

ROSE was highly concordant with pathological diagnosis (κ = 0.791; *P* < 0.001), with an accuracy of 95.7%. Diagnostic accuracy was significantly higher in the ROSE compared with the non‐ROSE group (96.3% vs 86.1%; *P* = 0.002), with overall incidences of complications of 36.8% and 23.8%, respectively. Minor pneumothorax without drainage was slightly greater in the ROSE compared with the non‐ROSE group (14.1% vs 6.6%; *P* = 0.046). However, there was no significant difference in serious complications between the two groups.

**Conclusion:**

ROSE was highly consistent with the final clinicopathological diagnosis for suspected lung cancer. ROSE further improved the diagnostic efficiency of CT‐guided TCNB with no increased incidence of serious complications.

## Graphical abstract

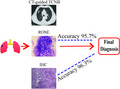

## 1 INTRODUCTION

Lung cancer is the leading cause of cancer‐related deaths worldwide.^1^ With the increase in low‐dose and high‐resolution computed tomography (CT) scanning for lung cancer, a large number of pulmonary lesions are inadvertently identified. However, only a few of those lesions are diagnosed as neoplasms and require further therapy. The pathological result is a standard criterion for diagnosis of lung cancer. Invasive interventions, such as bronchoscopy, transthoracic needle aspiration and surgical biopsy, are commonly used to acquire samples for pathological diagnosis. The samples of peripheral lung lesions are usually difficult to acquire by bronchoscopy, and are preferentially processed with transthoracic needle biopsy under the guidance of CT or ultrasound. CT‐guided transthoracic needle biopsy mainly includes core needle biopsy (CNB) and fine needle aspiration biopsy (FNAB). As higher volumes of tissue can be acquired, CNB improves the diagnostic yield in contrast to FNAB.^2^, ^3^, ^4^ A retrospective study showed that the diagnostic yield of CT‐guided transthoracic CNB (TCNB) was 91%.^2^ However, there are still some patients with lung cancer for whom there is a failure to obtain enough qualified samples for accurate diagnosis through CT‐guided TCNB, resulting in missed diagnosis and delay of effective therapy. Many efforts have been made to improve the diagnostic efficiency of peripheral lung lesions.

Rapid on‐site evaluation (ROSE) allows rapid cytomorphological evaluation and quick assessment of the adequacy and features of an acquired sample, which helps guidance for further biopsy. ROSE performed by an experienced pathologist or cytologist during FNAB has been reported to improve the diagnostic yield for lung and mediastinal lesions.^5^, ^6^, ^7^, ^8^ In previous studies the combination of imprint cytology and histology improved the diagnostic accuracy of CT‐guided transthoracic needle biopsy.^9^, ^10^, ^11^ However, data supporting the usage of ROSE during CT‐guided TCNB are limited, as previous studies were small scale or lacked a comparison with non‐ROSE assessment. This study was conducted to evaluate the diagnostic efficiency and safety of ROSE combined with CT‐guided TCNB for suspected lung tumour lesions in comparison to CT‐guided TCNB alone. In addition, the consistency between ROSE findings and the final clinicopathological diagnosis was investigated.

## 2 MATERIALS AND METHODS

### 2.1 Study population and procedure

**FIGURE 1:**
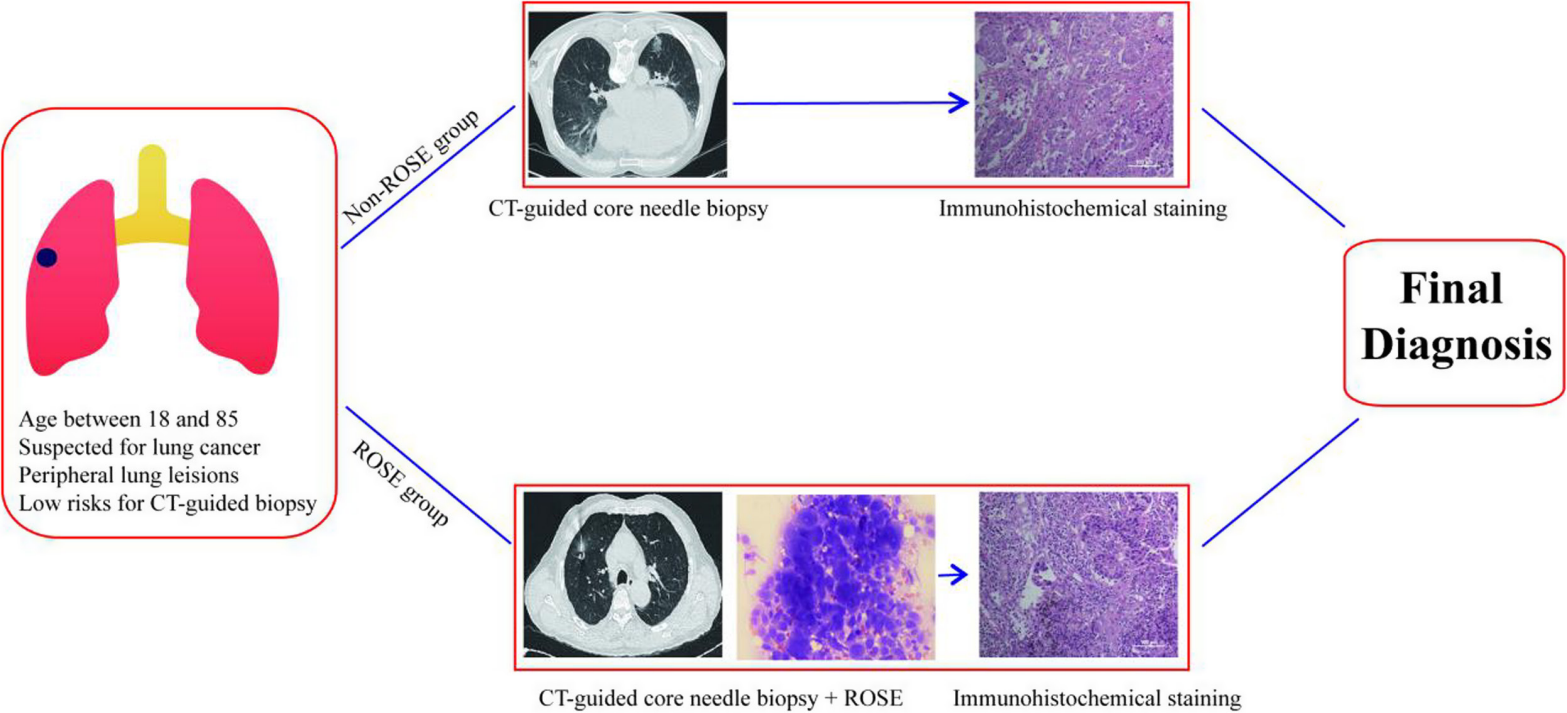
Inclusion and exclusion criteria and experimental design flow chart of this study

All patients with suspected lung cancer who underwent CT‐guided TCNB in the Respiratory Department of the Baoshan District of Huashan Hospital from January 2015 to December 2018 were included. Patients in this retrospective study were divided according to the usae of ROSE into a ROSE group and a non‐ROSE group. Suspected lung cancer patients receiving only CT‐guided TCNB between January 2015 and July 2016 were placed into the non‐ROSE group (*n* = 122). As the ROSE technique was subsequently adopted in our department, suspected lung cancer patients between August 2016 and December 2018 received ROSE during CT‐guided TCNB and were included in ROSE group (*n* = 163). The inclusion criteria for CT‐guided TCNB for patients in both groups were as follows: (a) age >18 years and <85 years; (b) having highly suspected lung cancer according to clinical features and chest imaging; (c) having lesions located in the periphery of lung, which are difficult to be acquired by bronchoscopy, or having a failure of diagnosis by previous transbronchial lung biopsy; (d) being at low risk for CT‐guided TCNB associated complications, that is, without severe emphysema or pulmonary bullae, uncontrolled cardiovascular disease, obvious bleeding tendency, or inability to cooperate with the procedure. The flow diagram of the study design is summarised in Figure 1. The diagnostic efficiency and complications of CT‐guided TCNB with or without ROSE were analysed, and the diagnostic yield of ROSE was compared with the pathological result of biopsy.

### 2.2 CT‐Guided transthoracic core needle biopsy (TCNB)

All included patients were fully informed of the benefits and risks of the biopsy and signed an informed consent form before the procedure. The operation of CT‐guided TCNB for pulmonary lesions was similar to that of a previous study.^12^ In brief, patients were positioned prone, supine, or lateral on the basis of the location of lesion on chest imaging. The entry site on the skin and the needle path were determined by CT images with the assistance of laser lighting and series of steel bars applied to the skin. After local anaesthesia, a 15‐guage coaxial guiding needle (Bard® TruGuide) was inserted into the thorax as planned by the interventional pulmonologist, followed by repeated CT scanning to confirm the path of needle toward the target lesion without serious complications or possible damage to important organs or vessels. Then, the automatic core biopsy instrument with a 16‐guage core needle (Bard® MAX‐CORE Disposable Core Biopsy Instrument) was placed into the coaxial needle, after removal of the stylet, to cut the tissue. In the ROSE group, each specimen was lightly rolled on 3 to 4 glass slides, one after another, and processed on‐site with Diff‐Quick staining by a cytologist. A rapid cytomorphological evaluation was performed under light microscope. The preliminary result about the adequacy of specimen and the morphological evaluation were obtained, which indicated a possible diagnosis of malignant or benign disease. If ROSE failed to make a preliminary diagnosis, another biopsy was to be performed until a positive diagnosis was made or severe complication occurred, or up to 5 sessions of biopsy. The samples in the non‐ROSE group and the remaining samples in the ROSE group were processed with haematoxylin and eosin staining, routine immunohistochemistry, and special staining (such as PAS staining) according to clinical requirements. Each patient underwent a further chest X ray to ascertain the occurrence of complications after 48 hours, or when symptoms associated with complications appeared.

### 2.3 Statistical analysis

SPSS software version 20.0 was used for data analysis. Continuous variables were expressed as medians interquartile ranges. Dichotomous variables were analysed using Pearson's chi‐squared test or Fisher’s exact test. Sensitivity, specificity, as well as positive and negative predictive values of CT‐guided TCNB with or without ROSE were calculated with a 2 × 2 table. The consistency between ROSE and the histological diagnosis was analysed with the Kappa statistic. A *P* value of <0.05 was considered to indicate a statistically significant difference.

## 3 RESULTS

### 3.1 Clinical characteristics of the patients

The clinical characteristics of the included patients are summarised in Table 1. In total, 285 patients were enrolled in this study, with 163 (57.2%) in the ROSE group. The ROSE and non‐ROSE group patients averaged 63.0 and 64.5 years in age, and included 108 (66.3%) and 85 (69.7%) males, respectively. The final clinicopathological diagnosis was made according to the pathology of TCNB, further surgical findings, aetiological examinations, and clinical follow‐up if required. The final clinicopathological diagnoses of included patients were as follows: 174 adenocarcinomas, 44 squamous carcinomas, 19 small cell lung cancers, 1 adenosquamous carcinoma, 11 non‐small cell lung cancers not otherwise specified, 4 undifferentiated carcinomas, 12 metastatic tumours, 1 malignant pleural mesothelioma, and 4 lymphomas. The remaining 15 patients were diagnosed with benign diseases, including 9 tuberculosis infections, 2 fungal infections, 2 bacterial pneumonia, 1 organising pneumonia, and 1 chronic inflammatory disease of non‐specific classification. There were no significant differences found in the analysed characteristics, including gender, age, size of biopsied lesions, and the final diagnoses of diseases, between those two groups (*P* > 0.05 for all analyses).

**TABLE 1:** Clinical characteristics of patients

Characteristics	ROSE group (*n* = 163)	Non‐ROSE group (*n* = 122)	*P* value
Gender, *n* (%)			0.512
Male	108 (66.26%)	85 (69.67%)	
Age, years (IQR)	63 (56–69)	64.5 (58–71)	0.294
Final clinicopathological diagnosis			
Diagnostic for malignancy	157	113	0.167
Adenocarcinoma	108	66	
Squamous carcinoma	21	23	
Small cell lung cancer	12	7	
Adenosquamous carcinoma	1	0	
NSCLC, not otherwise specified	4	7	
Undifferentiated carcinoma	4	0	
Metastatic malignancy	6	6	
Malignant pleural mesothelioma	0	1	
Lymphoma	1	3	
Benign outcome	6	9	
Size			
<3 cm	36	23	0.505
≥3 cm	127	99	

Abbreviations: IQR, interquartile range; NSCLC, non‐small cell lung cancer; ROSE, rapid on‐site evaluation.

### 3.2 High consistency between ROSE and final clinicopathological diagnosis

Comparison of the ROSE findings with the final clinicopathological diagnoses in 163 patients is shown in Table 2. A discrepancy between the ROSE evaluation and the final clinicopathological diagnosis was found in only 3 patients, who were preliminarily considered to be benign on ROSE but finally diagnosed as malignant according to pathology. The diagnostic sensitivity and specificity of ROSE were 95.5% and 100%, while the positive and negative predictive values were 100% and 66.7%, respectively. In addition, the diagnostic accuracy of ROSE was as high as 95.7% in our study. Of note, a high consistency was achieved between the ROSE result and the final clinicopathological diagnosis in suspected lung cancer patients (κ = 0.612; *p* < 0.001).

**TABLE 2:** Comparison between ROSE and the final clinicopathological diagnosis

ROSE	Final clinicopathological diagnosis		Total
	Benign	Malignant	
Benign	6	3	9
Malignant	0	150	150
Suspicious	0	4	4
Total	6	157	163

*Note*: Diagnostic sensitivity was 95.54%; Diagnostic specificity was 100%; Positive predictive values was 100%; Negative predictive value was 66.7%; Diagnostic accuracy was 95.71%

Abbreviation: ROSE, rapid on‐site evaluation.

### 3.3 ROSE combined with CT‐guided TCNB achieves high diagnostic efficiency

The final diagnosis confirmed the CT‐guided TCNB result in the ROSE group for 157/163 (96.3%) patients, with the other six patients finally diagnosed as malignancies being initially undiagnosed for lung cancer because of chronic inflammation (3 patients) and not having enough tumour cells (3 patients; Table 3). In the non‐ROSE group, patients with a confirmed diagnosis accounted for 86.1% (105/122), while 17 (13.9%) patients failed to be correctly diagnosed on CT‐guided TCNB alone. The incorrect diagnoses of these 17 non‐ROSE patients were as follows: 9 with chronic inflammation, 5 suspected tumour cells, 2 with necrosis, and 1 suspected tumour cell with necrosis. However, these 17 patients were ultimately diagnosed with lung cancer (13), metastatic tumours (2), lymphoma (1), and tuberculosis (1). The 23 initially undiagnosed patients by TCNB in the ROSE and non‐ROSE groups were confirmed by secondary percutaneous pulmonary biopsy or tracheoscopy. Compared with CT‐guided TCNB alone, ROSE combined with CT‐guided TCNB dramatically improved the diagnostic accuracy from 86.1% to 96.3% in patients with suspected lung cancer (*P* = 0.002).

**TABLE 3:** Comparison of tissue diagnostic value

Group	Final clinicopathological diagnosis		Total
	Confirmation/support	Suspicious/failure	
ROSE	157	6	163
Non‐ROSE	105	17	122
Total	262	23	285

Abbreviation: ROSE, rapid on‐site evaluation.

### 3.4 ROSE combined with CT‐guided TCNB shows no increased risk of severe complication

As shown in Table 4, biopsy procedure‐associated complications were reported in 60 patients (36.8%) in the ROSE group and 29 patients (23.8%) in the non‐ROSE group (χ^2^ = 5.524; *P* = 0.019). Pneumothorax was the most common complication, which occurred in 20.9% and 13.1% patients in the ROSE and non‐ROSE groups, respectively (*P* = 0.089). Mild pneumothorax (less than 30% of lung compression) without thoracic drainage slightly increased in the ROSE group compared with the non‐ROSE group (14.1% vs 6.6%; *P* = 0.043). However, the incidence of severe pneumothorax requiring thoracic drainage was similar between the two groups (6.8% vs 6.6%; *P* = 0.949). Haemorrhage occurred in 21/163 (12.9%) patients in the ROSE group and 11/122 (9.0%) patients in the non‐ROSE groups, respectively, with no significant difference (*P* = 0.306). Only one patient in the ROSE group developed haemothorax requiring thoracic drainage. Other rare complications, including pleural reaction, chest distress, and subcutaneous emphysema, showed no significant difference between these two groups (*P* = 0.638). It was noteworthy that none of those patients experienced life‐threatening complications.

**TABLE 4:** Comparison of CT‐guided CNB associated complications

Complications	ROSE group (N = 163)	Non‐ROSE group (N = 122)	*p* value
Pneumothorax	34	16	0.089
<30%	23	8	
≥30%	11	8	
Haemoptysis	21	11	0.306
Mild	21	11	
Moderate/severe	0	0	
Haemothorax	1	0	1.000^a^
Others	4	2	1.000^a^

Abbreviations: CT‐guided CNB, computed tomography‐guided core needle biopsy; ROSE, rapid on‐site evaluation.^a^Fisher exact test.

## 4 DISCUSSION

An adequate qualified biopsy sample is essential for confirming a diagnosis of lung cancer. According to the recommendations in the latest National Comprehensive Cancer Network guideline, histological confirmation is required for lung cancer before any non‐surgical therapy. CT‐guided TCNB is an effective method for the diagnosis of peripheral lung lesions. However, some atypical histological findings may affect the final diagnosis. A small portion of patients may fail to achieve definite diagnosis by CT‐guided TCNB, which could delay the treatment. In addition, repeated biopsy may increase the risk of procedure‐associated complications due to its invasive nature. It is particularly important to improve diagnostic efficiency and guarantee patients' safety as well. In this study, we retrospectively evaluated the efficiency and safety of ROSE combined with CT‐guided TCNB for the diagnosis of patients with suspected lung cancer.

ROSE offers timely feedback regarding the cytomorphological adequacy and character of the biopsy sample, in addition to a preliminary diagnosis of the lesion, and is commonly used to improve diagnostic efficiency for lung lesions.^13^, ^14^ In our study, the diagnostic sensitivity and specificity of ROSE were both greater than 95%. Meanwhile, the positive predictive value and negative predictive value were 100% and 66.7%, respectively, with the diagnostic accuracy of ROSE as high as 95.7%. These results are consistent with those of a previous study that found improved diagnostic yield was achieved by ROSE during transbronchial biopsy for peripheral lung cancer.^15^

CT‐guided TCNB is a commonly used technique to obtain tissue from lesions located in the periphery of the lung for differential diagnosis. As reported in previous studies, the diagnostic accuracy of CT‐guided TCNB ranged from 83.4% to 97.6%.^16^, ^17^, ^18^ A preliminary study containing 94 lesions indicated that ROSE combined with CT‐guided transthoracic lung biopsy achieved a diagnostic yield of 94.7%, which was higher than either histological diagnosis (91.5%) or cytological evaluation (83.0%) alone.^10^ The results of our study showed a higher diagnostic accuracy of 96.3% in ROSE group than that of 86.1% in the non‐ROSE group (*P* = 0.002). These findings indicate that the combined usage of ROSE with CT‐guided TCNB can achieve a better diagnostic yield for peripheral lung lesions than CT‐guided TCNB alone.

As reported in a meta‐analysis, the pooled complication rate for CT‐guided transthoracic TCNB was 38.2%, whereas the incidence of main complications ranged from 4.8% to 5.7%.^19^ Pneumothorax is the most common complication for transthoracic needle biopsy, with an incidence of 12%–45%.^20^ The rate of thoracic catheterisation to relieve the symptoms of biopsy‐associated complications varied greatly across studies, and was reported to be 2.0%–15.0%.^20^ Incidence of rare complications for CT‐guided TCNB was found to be 0.02%–0.4% for air embolism,^20^, ^21^ 0.012% for needle tract tumour seeding^22^ and 0.16% for death.^23^ However, none of these rare complications just mentioned were reported in our study. The risk factors for the complications of CT‐guided TCNB included longer needle path length from pleura to the lesion, emphysema, numbers of lesions, lesion located in lower lobe, small lesion, and supine position.^16^, ^19^, ^24^ In our study, the overall incidence rate of complications in the ROSE group was slightly higher than that in the non‐ROSE group, mainly presented as the increased occurrence of mild pneumothorax without drainage. Patients with mild pneumothorax required oxygen therapy only, which might have resulted from repeated adjustment of the needle and increased sessions of biopsy in the ROSE group. The occurrence of other complications, including major pneumothorax requiring drainage, pulmonary haemorrhage, and some rare complications, was similar between the two groups. In addition, it was noteworthy that no life‐threatening complication associated with biopsy occurred in this study.

This was the first study to compare the efficiency and safety of the combined use of ROSE and CT‐guided TCNB versus CT‐guided TCNB alone in the diagnosis of peripheral lesions of the lung in a clinical study. However, a few limitations need to be considered regarding this study. First, the risk factors associated with increased complications, such as emphysema, length of needle path from pleura to the lesion, and operation time, were not analysed. Second, the retrospective nature of the data might have caused selection and recall bias or inconsistencies.

## 5 CONCLUSION

The results of CT‐guided TCNB with ROSE were highly concordant with the final clinicopathological diagnosis for suspected malignant lesions of the peripheral lung. Of note is that the accuracy of ROSE reached as high as 95.7% for suspected lung cancer lesions. ROSE combined with CT‐guided TCNB improved the diagnostic efficiency without increasing the incidence of major complications associated with biopsy. In summary, the combination of ROSE with CT‐guided TCNB was an effective and safe approach for the diagnosis of potential malignant lesions located in the periphery of the lung.

## CONFLICTS OF INTEREST

All authors have completed the ICMJE uniform disclosure form. The authors have no conflicts of interest to declare.

## AUTHOR CONTRIBUTIONS

Conception and design: All authors. Administrative support: RY, ZXJ, LSQ. Provision of study materials or patients: RY, ZXJ, CK, ZYY. Collection and assembly of data: RY, ZN, LCW, ZDB. Data analysis and interpretation: ZXJ, LJ, ZYH, LSQ. Manuscript writing: All authors. Final approval of manuscript: All authors.

## ETHICAL APPROVAL

This study was approved by the Institutional Review Board of Huashan Hospital, affiliated with Fudan University (Permit Number: KY2016‐396). In this retrospective study, the data are anonymous, and the requirement for informed consent was therefore waived.

## Data Availability

The data that support the findings of this study are available from the corresponding author upon reasonable request.

## References

[1] Siegel RL , Miller KD , Jemal A . Cancer statistics, 2020. CA Cancer J Clin. 2020;70:7‐30. 31912902 10.3322/caac.21590

[2] Sangha BS , Hague CJ , Jessup J , O'Connor R , Mayo JR . Transthoracic computed tomography‐guided lung nodule biopsy: comparison of Core needle and fine needle aspiration techniques. Can Assoc Radiol J. 2016;67:284‐289. 27005931 10.1016/j.carj.2015.10.005

[3] Capalbo E , Peli M , Lovisatti M , et al. Trans‐thoracic biopsy of lung lesions: FNAB or CNB? Our experience and review of the literature. Radiol Med. 2014;119:572‐594. 24297594 10.1007/s11547-013-0360-1

[4] Beslic S , Zukic F , Milisic S . Percutaneous transthoracic CT guided biopsies of lung lesions; fine needle aspiration biopsy versus core biopsy. Radiol Oncol. 2012;46:19‐22. 22933975 10.2478/v10019-012-0004-4 PMC3423761

[5] Xu CH , Wang JW , Wang W , et al. The diagnosis value of endobronchial ultrasound transbronchial lung biopsy combined with rapid on‐site evaluation in peripheral lung cancer. Clin Respir J. 2020;14:447‐452. 31916391 10.1111/crj.13151

[6] Madan K , Dhungana A , Mohan A , et al. Conventional Transbronchial needle aspiration versus Endobronchial ultrasound‐guided Transbronchial needle aspiration, with or without rapid on‐site evaluation, for the diagnosis of Sarcoidosis: a randomized controlled trial. J Bronchology Interv Pulmonol. 2017;24:48‐58. 27984385 10.1097/LBR.0000000000000339

[7] Mazza E , Maddau C , Ricciardi A , Falchini M , Matucci M , Ciarpallini T . On‐site evaluation of percutaneous CT‐guided fine needle aspiration of pulmonary lesions. A study of 321 cases. Radiol Med. 2005;110:141‐148. 16200036

[8] Umeda Y , Otsuka M , Nishikiori H , et al. Feasibility of rapid on‐site cytological evaluation of lung cancer by a trained pulmonologist during bronchoscopy examination. Cytopathology. 2019;30:628‐633. 31479551 10.1111/cyt.12771

[9] Chang YC , Yu CJ , Lee WJ , et al. Imprint cytology improves accuracy of computed tomography‐guided percutaneous transthoracic needle biopsy. Eur Respir J. 2008;31:54‐61. 17928311 10.1183/09031936.00038907

[10] Yamagami T , Yoshimatsu R , Kajiwara K , et al. Effectiveness of combined use of imprint cytological and histological examination in CT‐guided tissue‐core biopsy. Eur Radiol. 2014;24:1127‐1134. 24526285 10.1007/s00330-014-3104-2

[11] Prosch H , Hoffmann E , Bernhardt K , et al. Dynamic telecytologic evaluation of imprint cytology samples from CT‐guided lung biopsies: a feasibility study. Eur Radiol. 2011;21:1922‐1927. 21553316 10.1007/s00330-011-2137-z

[12] Li Y , Du Y , Yang HF , et al. CT‐guided percutaneous core needle biopsy for small (≤20 mm) pulmonary lesions. Clin Radiol. 2013;68:e43‐e48. 23177650 10.1016/j.crad.2012.09.008

[13] Xu C , Wang W , Yuan Q , Hu H , Li L , Yang R . Rapid on‐site evaluation during radial Endobronchial ultrasound‐guided Transbronchial lung biopsy for the diagnosis of peripheral pulmonary lesions. Technol Cancer Res Treat. 2020;19:1533033820947482. 32812488 10.1177/1533033820947482 PMC7440722

[14] Shikano K , Ishiwata T , Saegusa F , et al. Feasibility and accuracy of rapid on‐site evaluation of touch imprint cytology during transbronchial biopsy. J Thorac Dis. 2020;12:3057‐3064. 32642228 10.21037/jtd-20-671 PMC7330746

[15] Wang J , Zhao Y , Chen Q , et al. Diagnostic value of rapid on‐site evaluation during transbronchial biopsy for peripheral lung cancer. Jpn J Clin Oncol. 2019;49:501‐505. 30855687 10.1093/jjco/hyz025

[16] Huang MD , Weng HH , Hsu SL , et al. Accuracy and complications of CT‐guided pulmonary core biopsy in small nodules: a single‐center experience. Cancer Imaging. 2019;19:51. 31337425 10.1186/s40644-019-0240-6 PMC6651998

[17] Tongbai T , McDermott S , Kiranantawat N , et al. Non‐diagnostic CT‐guided percutaneous needle biopsy of the lung: predictive factors and final diagnoses. Korean J Radiol. 2019;20:1515‐1526. 31606956 10.3348/kjr.2019.0014 PMC6791813

[18] Porrello C , Gullo R , Gagliardo CM , et al. CT‐guided transthoracic needle biopsy: advantages in histopathological and molecular tests. Future Oncol. 2020;16:27‐32. 31596139 10.2217/fon-2019-0076

[19] Heerink WJ , de Bock GH , de Jonge GJ , Groen HJM , Vliegenthart R , Oudkerk M . Complication rates of CT‐guided transthoracic lung biopsy: meta‐analysis. Eur Radiol. 2017;27:138‐148. 27108299 10.1007/s00330-016-4357-8 PMC5127875

[20] Gupta S , Wallace MJ , Cardella JF , et al. Quality improvement guidelines for percutaneous needle biopsy. J Vasc Interv Radiol. 2010;21:969‐975. 20304676 10.1016/j.jvir.2010.01.011

[21] Tomiyama N , Yasuhara Y , Nakajima Y , et al. CT‐guided needle biopsy of lung lesions: a survey of severe complication based on 9783 biopsies in Japan. Eur J Radiol. 2006;59:60‐64. 16530369 10.1016/j.ejrad.2006.02.001

[22] Ayar D , Golla B , Lee JY , Nath H . Needle‐track metastasis after transthoracic needle biopsy. J Thorac Imaging. 1998;13:2‐6. 9440831 10.1097/00005382-199801000-00002

[23] Freund MC , Petersen J , Goder KC , Bunse T , Wiedermann F , Glodny B . Systemic air embolism during percutaneous core needle biopsy of the lung: frequency and risk factors. BMC Pulm Med. 2012;12:2. 22309812 10.1186/1471-2466-12-2 PMC3608336

[24] Tsou MH , Tsai SF , Chan KY , et al. CT‐guided needle biopsy: value of on‐site cytopathologic evaluation of core specimen touch preparations. J Vasc Interv Radiol. 2009;20:71‐76. 19028112 10.1016/j.jvir.2008.10.011

